# Simultaneous bidirectional hindlimb locomotion in decerebrate cats

**DOI:** 10.1038/s41598-021-82722-2

**Published:** 2021-02-05

**Authors:** V. Lyakhovetskii, N. Merkulyeva, O. Gorskii, Pavel Musienko

**Affiliations:** 1 Russian Research Center of Radiology and Surgical Technologies, Ministry of Healthcare of the RF, Poselok Pesochnyy, Leningradskaya st., 70, Saint-Petersburg, Russia 197758; 2 Pavlov Institute of Physiology, Russian Academy of Sciences, emb. Makarova 6, Saint-Petersburg, Russia 199034; 3 Institute of Translational Biomedicine, Saint-Petersburg State University, Universitetskaya emb. 7/9, Saint-Petersburg, Russia 199034; 4 Children’s Surgery and Orthopedic Clinic, Saint-Petersburg State Research Institute of Phthisiopulmonology, Ministry of Healthcare of the RF, Saint-Petersburg, Russia 191036

**Keywords:** Central pattern generators, Spinal cord, Dynamical systems, Neurophysiology

## Abstract

We show that epidural spinal cord stimulation can elicit stable bidirectional locomotion of decerebrate cats on a split-belt treadmill. The stepping pattern of one limb was similar to unidirectional forward walking and, the other—was similar to unidirectional backward walking. This confirms that spinal and brainstem circuitry are sufficient to control such complex and extraordinary motor tasks driven by somatosensory input. Interlimb coordination during forward and backward walking was preserved in 2 out of 4 animals during ‘extreme’ conditions when one of the treadmill belts was stopped. Bidirectional locomotion worsened but was still possible after temporary spinalization by cooling the spinal cord on a low thoracic level. These present evidence for the great degree of the automatism for this stepping mode defined by the spinal neuronal networks.

## Introduction

All animals are able to coordinate the movements of their limbs. Such coordination is a complex multi-level process. Quadrupeds control the movements of each limb from the spinal cord level through the central pattern generator (CPG) located in the cervical and upper thoracic segments—for forelimbs^1^, and in the lower thoracic and lumbar segments—for hindlimbs^2,3^. According to the multi-level hypothesis, the CPG has different, partly independent, blocks for rhythm generation and pattern formation^4,5^.

Since the pioneer work of Kulagin and Shik^6^, the split-belt treadmill experimental design has been widely used for studying the mechanisms of left–right CPGs coordination^7–11^. The *speed* of each belt has varied independently during the *forward* walking (FW) of an intact^9^, spinal^8,9,12^ or decerebrate^11^ cat or in humans^10,13^, resulting in the unstable locomotor pattern during the first cycles of the stepping and in the subsequent adaptation. The ability of both hindlimbs to synchronize during split-belt locomotion^6,8,9,12^ has led to the assumption that the CPGs of the left and right limbs are connected through commissural interconnections^14^. Such interconnections may have complex structures including different types of commissural interneurons^15,16^.

Meanwhile, *bidirectional* (BIDI) locomotion, in which one limb performs forward locomotor movements while the other simultaneously steps backward, may be induced in healthy infants^17^ and adult humans^7^. In nature this mode of locomotion may be used by an animal while turning around its own axis. A similar pattern of hindlimb movement in mixed-form swimming was described in intact red-eared turtles^18^. It is not known, however, if the spinal cord and brainstem circuitry were able to accomplish simultaneous bidirectional hindlimb locomotion or if conscious control by the forebrain is necessary for this task.

Thus, we used the split-belt treadmill to study the bilateral coordination during BIDI stepping in decerebrate cats. The parameters of this locomotor mode were compared with the parameters of the unidirectional FW and backward walking (BW). Each locomotor mode was elicited in the same manner by the electrical epidural stimulation (ES) of the lumbar spinal cord.

A brief account of a part of this study was previously published in abstract form^19^.

## Materials and methods

### Subjects

Seven normal pigmented male adult cats (weighing 2.5–3.5 kg) reared in the Pavlov Institute of Physiology breeding colony were used. All experimental procedures were approved by the Ethics Commission of the Pavlov Institute of Physiology. Experiments were performed in accordance with the requirements of Council Directive 2010/63EU of the European Parliament on the protection of animals used in experimental and other scientific purposes.

### Surgical procedures and electrical stimulation

**Figure 1:**
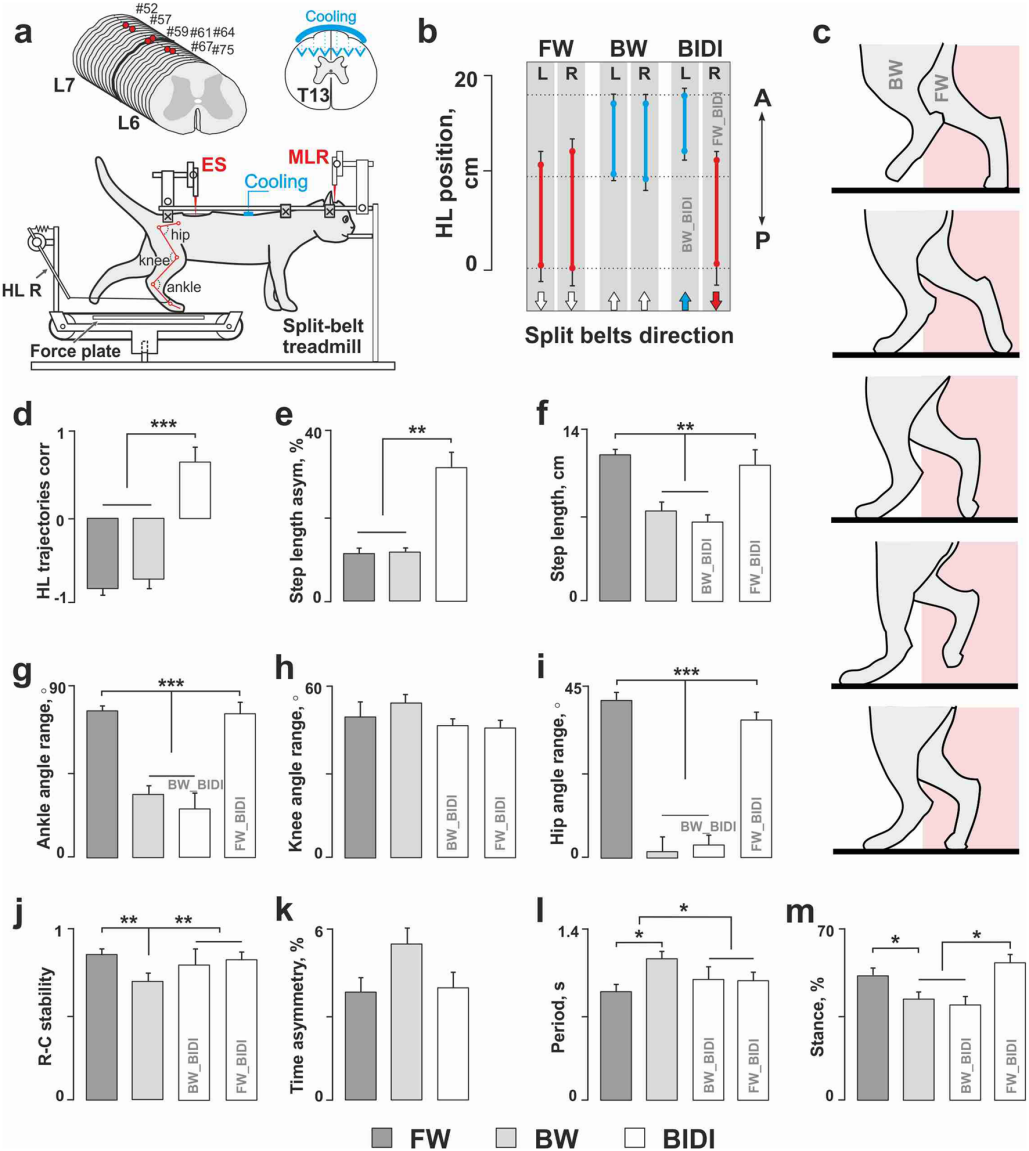
Kinematics of the bidirectional BIDI stepping in comparison to unidirectional FW and BW. (**a**) Experimental design. Walking was elicited by the electrical epidural stimulation (ES) of the dorsal surface of L6 or L7 spinal segments and stimulation of the mesencephalic locomotor region (MLR). Anterior/posterior movements of each hindlimb were recorded by a sensor (only the right sensor, HL R, is shown). Ground reaction forces (GRF) were recorded by the force sensors under the left and right belt. Red circles indicate the reflective markers attached to the skin in projections of the main hindlimb joints. The cooling of the spinal cord for temporary spinalization was done on the T13 segment. (**b**) The positions (mean ± SE, n = 7 cats) of the left (L) and right (R) hindlimbs on the treadmill belts at FW, BW and BIDI stepping, A, P—anterior–posterior axis. (**c**) Positions of hindlimbs during equally spaced phases of BIDI step cycle. The pink rectangles mark the treadmill area occupied by hindlimb moving forward. (**d**,**e**) Correlation of hindlimb trajectories and step length asymmetry at FW, BW and BIDI stepping. (**g**–**j**) step length, angle range in ankle, knee and hip respectively and rostro-caudal stability at FW, BW and BIDI stepping (BW_BIDI means hindlimb moving backward, FW_BIDI means hindlimb moving forward during BIDI stepping). The rostro-caudal stability of locomotor movements of individual limb was estimated using the self-similarity coefficient. (**k**–**m**) Timing of the BIDI stepping in comparison to unidirectional FW and BW. (**k**) The asymmetry of step cycle period. (**L**) The step cycle period. (**m**) The mean proportion of stance phase in a step cycle. (**d**–**m**) Significance level: *p < 0.05, **p < 0.01, ***p < 0.001, mean ± SE, n = 7 cats.

The common surgical procedures were described in detail in^20,21^. Shortly, the deep anesthesia was performed with a mixture of Isoflurane (2–4%) and Oxygen during the surgical procedures. The animals were decerebrated at the precollicular–postmammilar level. Then the head of the animal, the vertebral column, and the pelvis were fixed in a rigid frame (Fig. 1a). The distance between the treadmill belt and the pelvis was 21–25 cm. The forelimbs were hanging in the air. Electromyograms (EMGs) from selected muscles were recorded using bipolar electromyographic electrodes (0.2 mm flexible stainless-steel Teflon-insulated wires) implanted into m. iliopsoas (IP, hip flexor), m. tibialis anterior (TA, ankle flexor), m. gastrocnemius medialis (GM, ankle extensor) of both hindlimbs. The rectal temperature, electrocardiographic and breathing rates were continuously monitored.

Since we obtained previously that BW can be induced by ES of the narrow region of the spinal cord (segments L5–L7)^21^, to induce BIDI stepping we stimulate just this area preliminary determined in a given animal. A monopolar silver ball electrode (d = 0.5 mm) was positioned 2–3 h after the end of surgical procedures on the optimal point of the dura mater of the spinal cord dorsal surface to stably evoke all three modes of locomotion by ES. The same location and parameters of stimulation per animal was used to induce BW, FW and BIDI stepping (Fig. 1a). The precise identification of the stimulating point was determined post-mortem on the base of interroot-root variant of spinal cord segmental division^22^. Similarly to Merkulyeva et al.^21^ we used the following parameters of stimulation: frequency, 5 Hz; pulse duration, 0.2–0.5 ms. The current intensity was varied from one animal to another and consisted of 80–300 μA. The hindlimbs were positioned on the separate treadmill belts moving either both backward (for unidirectional FW), or both forward (for unidirectional BW)^23,24^, or in opposite directions (for BIDI locomotion). The speed of both belts was equal to 0.45 m/s for all observed types of locomotion. The treadmill started to move 2–3 s before ES began. To check the strength of the commissural connectivity, we tested the ability of animals (n = 4) to locomote at ‘extreme’ conditions by stopping one of the treadmill belts.

To simulate the *spinalization* for studying the supraspinal influence onto the BIDI locomotion, a temporary cooling of the spinal cord dorsal surface was performed with one animal using the modified approach described previously^25^. The cooler was connected to the metal half-round concave tip contacting the dorsal surface of the T13 spinal cord segment. The temperature measured onto the dorsal surface of the spinal cord decreased to the desired after the cooler was switched on, and thereafter increased up to the normal level (+ 37 °C). Both the cooling up to the temperature of interest and the heating lasted for several seconds. During cooling of the spinal cord, the animal's ability of BIDI stepping was quickly tested (for 1–2 min). To test the efficacy of the blockade of the supraspinal descending commands during cooling, the electrical stimulation of the mesencephalic locomotor region (MLR), one of the brainstem centers specifically dedicated to initiate and control locomotion^26^, was performed. Bipolar tungsten electrode was inserted into the brainstem area (Horsly–Clark coordinates P2, R/L4, H0) by means of a micromanipulator (Fig. 1a). A 30 Hz stimulation (0.1 ms duration, 30–50 µA current) was applied.

### Analysis and statistics

To characterize kinematics of locomotor movements, reflective markers were placed on the iliac crest, femoral head, lateral condyle of the femur, lateral malleolus, fifth metatarsal joint, and the side view of the walking cat was video recorded (25 frames/s). To compare kinematics at different locomotor modes, the video recordings were analyzed frame-by frame. The angles were calculated at the moment when the hindlimb was maximally flexed during the swing phase (Swing angles) and at the moment when it was maximally extended (Stance angles) in the stance phase^21^. The angle ranges were calculated as the difference between the stance and swing angles for each joint. In addition, we recorded the anterior/posterior (A–P) limb movements (by means of two mechanical sensors attached to the ankles) and each limb ground reaction forces synchronized with video (Fig. 1a). The signals from the EMG electrodes and from the mechanical sensors were amplified and digitized at 20 kHz.

The correlation coefficient for hindlimb trajectories was calculated on the base of the time series of A-P hindlimb movements. As in Merkulyeva et al.^21^, the asymmetry of locomotor movements performed by the left and right hindlimbs was estimated with the help of coefficient of step length asymmetry calculated as |S_left_ − S_right_|/(S_left_ + S_right_), where S_left_ and S_right_ are the paw excursions of the left and right limb, respectively. The paw excursion was calculated as the distance between the position of the paw at the beginning and at the end of the stance phase. The coefficient of step cycle asymmetry was calculated in a similar way as |T_left_ − T_right_|/(T_left_ + T_right_), where T_left_ and T_right_ are the cycle periods of the left and right limb, respectively. As in Merkulyeva et al.^21^, the stability of locomotor movements of the individual limb in the rostrocaudal plane was estimated using the self-similarity coefficient (the amplitude of the autocorrelation functions second peak of the time series of A–P movements of the individual limb). The relative IP, TA and GM muscle activity was calculated as the ratio of maximum muscle activity during the BW or BIDI step cycle relative to the maximum muscle activity during the FW step cycle.

The data is presented as mean ± standard error. Statistical significance was assessed using the Wilcoxon sign-rank test; W(n) in the text below signifies the sum of ranks of test statistic W for n samples taken.

## Results

We found that all decerebrate cats studied (n = 7) were capable of simultaneous BIDI locomotion on a split-belt treadmill (Fig. 1a,b) during ES of the spinal cord. The initiation period, that is, the time after the start of ES^27^, of this locomotor mode was 1.7 ± 0.2 s. The stepping pattern of one hindlimb (FW_BIDI) was similar to unidirectional FW, and that of the other hindlimb (BW_BIDI) was similar to unidirectional BW elicited by ES with the same location and parameters (Fig. 1c, Supplement Video). We used a particular period of the ES-evoked locomotion, optimal for our conditions, that lasted for 20–30 s. During this period, the quality of unidirectional and bidirectional stepping did not change; that is, the rostrocaudal stability, the time asymmetry and other stepping characteristics remained unchanged. The maximum duration of the BIDI locomotion was 30 s.

### The general kinematic features of the BIDI locomotion

The FW_BIDI hindlimb was located more caudally than the BW_BIDI hindlimb during BIDI locomotion; the same ratio of positions was shown for unidirectional FW and BW (Fig. 1b). The two hindlimbs were moving almost in anti-phase for unidirectional FW and BW, resulting in a strong negative correlation coefficient between their trajectories, while for BIDI locomotion, the hindlimbs were moving almost in-phase resulting in a strong positive correlation coefficient between their trajectories (Fig. 1d). Thus, the correlation coefficients for the hindlimb trajectories were significantly different for the BIDI locomotion than for both unidirectional FW and BW (R_BIDI_ = 0.66 ± 0.11 vs. R_FW_ = − 0.82 ± 0.04, R_BW_ = − 0.71 ± 0.08, W(7) = 28, *p* = 0.0156). The BIDI locomotion pattern did not change during its period, however, the correlation coefficients for the hindlimb trajectories insignificantly increased in the second half of the trials in relation to the first half (0.72 ± 0.12 vs. 0.66 ± 0.12, W(7) = 24, *p* = 0.1094).

Both hindlimbs shared similar kinematic characteristics during unidirectional FW and BW. Conversely, the kinematic characteristics of the two limbs in BIDI mode were different and corresponded to the appropriate unidirectional mode. Thus, BIDI locomotion had pronounced step length asymmetry in relation to unidirectional FW and BW (31.15 ± 5.09% vs. 11.08 ± 1.43% and 11.35 ± 2.68%, W(7) = 28, *p* = 0.0156) (Fig. 1e), because the steps of the FW_BIDI hindlimb, similar to the steps during unidirectional FW, were longer than the steps of BW_BIDI hindlimb and unidirectional BW (11.12 ± 1.89 cm and 11.93 ± 1.01 cm vs. 6.47 ± 0.56 cm and 7.36 ± 0.48 cm, W(7) = 28, *p* = 0.0156) (Fig. 1f). Similarly, the ‘swing-stance’ angle ranges of the hip and ankle joints (Fig. 1g,i) showed similar pattern to limbs stepping FW/BW during unidirectional and bidirectional locomotion. The ‘swing-stance’ angle ranges in these joints were significantly higher for FW and FW_BIDI hindlimbs relative to BW and BW_BIDI hindlimbs (ankle: 73.64 ± 3.25° and 68.63 ± 7.53° vs. 26.03 ± 7.51° and 22.74 ± 4.64°, W(7) = 28, *p* = 0.0156; hip: 39.93 ± 2.62° and 34.54 ± 6.73° vs. 5.08 ± 0.89° and 6.34 ± 1.58°, W(7) = 28, *p* = 0.0156). The ‘swing-stance’ angle ranges in the knee joint did not differ between observed locomotor modes because the knee joint was equally as active during FW and BW (Fig. 1h).

The rostrocaudal stability of movements for both hindlimbs during BIDI stepping was similar to that during FW and was higher than during BW (0.79 ± 0.04, 0.82 ± 0.02 and 0.86 ± 0.01 vs. 0.69 ± 0.03, W(7) = 28, *p* = 0.0156) (Fig. 1j).

### Temporal characteristics of the step cycle

The asymmetry of the period of step cycles did not depend on the locomotor mode (Fig. 1k); the step cycles of FW_BIDI and BW_BIDI hindlimbs were approximately of equal duration. Meanwhile, the step cycle of FW was shorter compared than that of BW (0.90 ± 0.04 s vs. 1.16 ± 0.07 s, W(14) = 103, *p* = 0.0002). The step cycles of FW_BIDI and BW_BIDI hindlimbs were approximately equal and had intermediate values; they were longer than that of FW (1.00 ± 0.09 s and 0.98 ± 0.1 s vs. 0.90 ± 0.04 s, W(7) = 28, *p* = 0.0156) and were shorter than that of BW (1.00 ± 0.09 s and 0.98 ± 0.1 s vs. 1.16 ± 0.07 s, W(7) = 28, *p* = 0.0156) (Fig. 1l). At the same time, the notable differences in the internal structure of the step cycle were found for two hindlimbs during BIDI stepping. The proportion of the stance phase in a cycle for the FW_BIDI hindlimb was greater than for the BW_BIDI hindlimb (56.34 ± 1.97% vs. 39.05 ± 3.33%, W(7) = 28, *p* = 0.0156), similar to the difference between the unidirectional FW and BW themselves (51.15 ± 2.10% vs. 41.46 ± 2.87%, W(14) = 103, *p* = 0.003) (Fig. 1m).

### Muscle activity during step cycle

**Figure 2:**
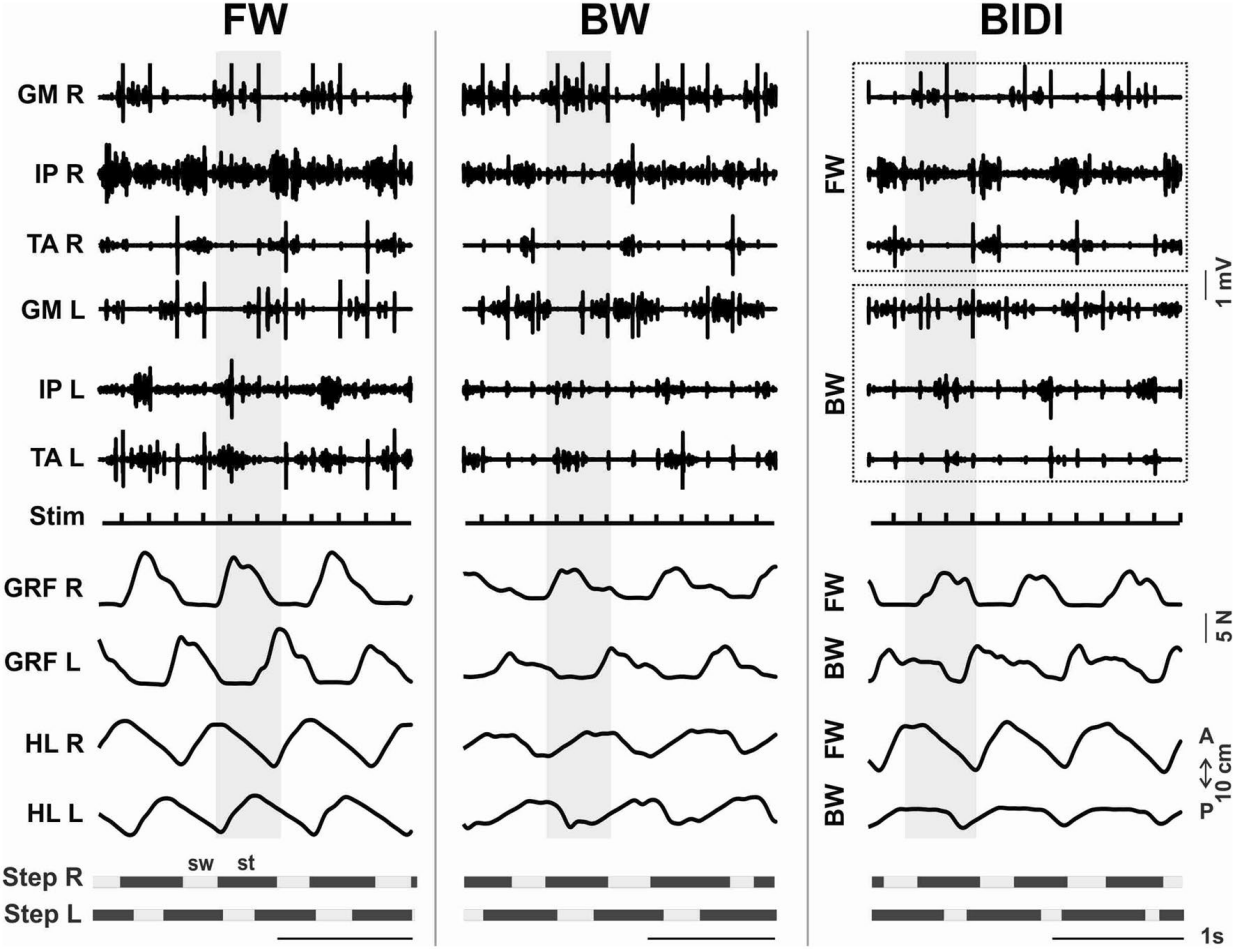
EMG pattern of the BIDI stepping in comparison to unidirectional FW and BW. Examples of left and right ground reaction forces (GRF L, GRF R) and EMG pattern of left and right mm. iliopsoas (IP), tibialis anterior (TA) and gastrocnemius (GM) during subsequent stepping of left and right hindlimbs (HL L, HL R). The muscle activity of hindlimbs moving forward and backward during BIDI are marked by dotted rectangles. Shaded gray areas marked the activity during one stance phase of the right hindlimb.

**Figure 3:**
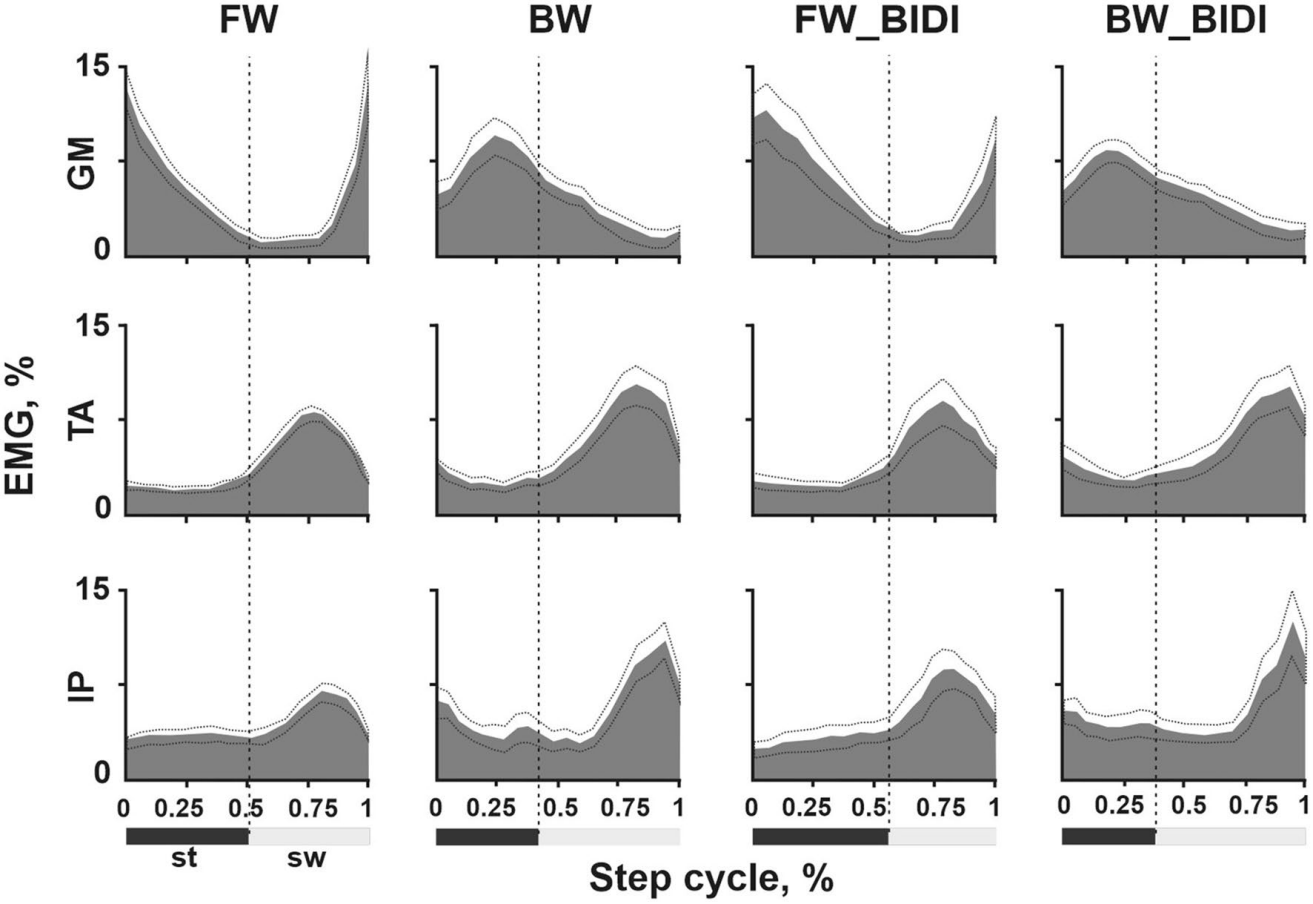
EMG pattern of gastrocnemius medialis (GM) (upper row), tibialis anterior (TA) (middle row) and iliopsoas (IP) (bottom row), during the FW, BW, FW_BIDI and BW_BIDI step cycle (BW_BIDI means hindlimb moving backward, FW_BIDI means hindlimb moving forward during BIDI stepping). EMG values were normalized to total cycle activity. Dotted line—a boundary between stance (st) and swing (sw) step phases. Mean ± SE, n = 7 cats.

**Figure 4:**
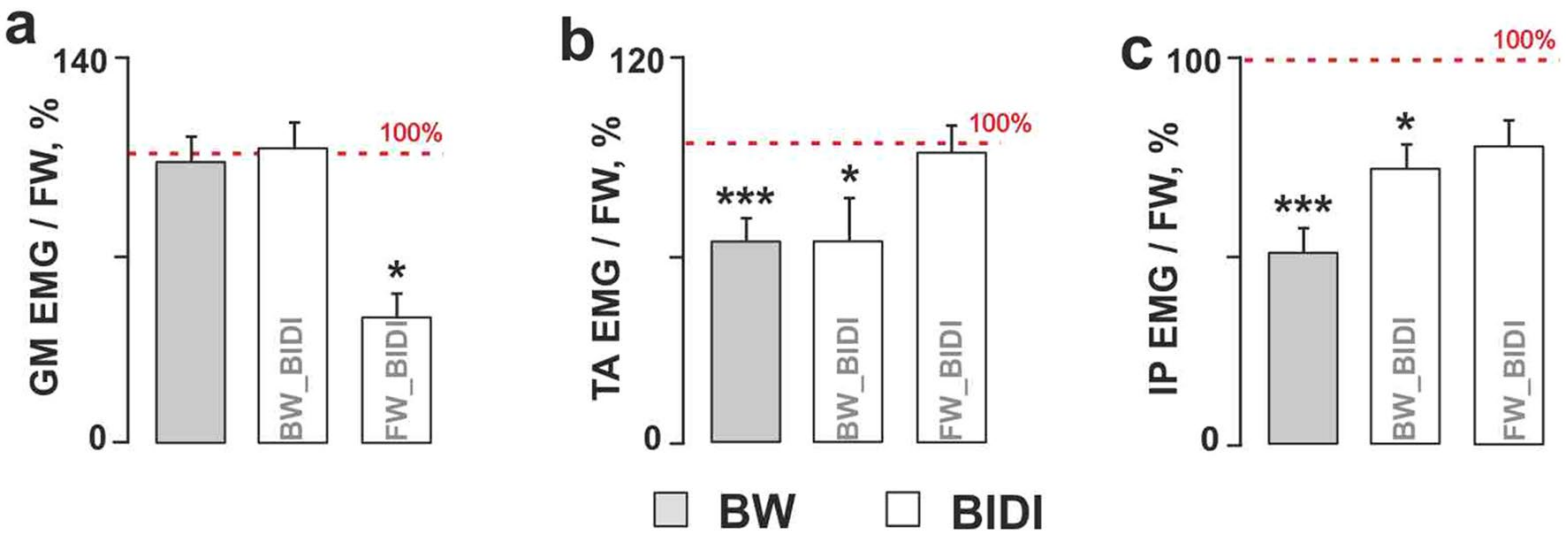
(**a**–**c**) The relative muscle activity calculated as the ratio of maximum muscle activity during BW or BIDI step cycle relative to the maximum muscle activity during FW step cycle (BW_BIDI means hindlimb moving backward, FW_BIDI means hindlimb moving forward during BIDI stepping). (**a**) Gastrocnemius medialis (GM). (**b**) Tibialis anterior (TA). (**c**) Iliopsoas (IP). Significant difference from FW mode or between the conditions: * p < 0.05, ***p < 0.001**,** mean ± SE, n = 7 cats.

We found that the high level of stability of the hindlimb movements during BIDI stepping described above was accompanied by stable reciprocal bursting activity of the ipsilateral antagonistic muscles (TA and GM), specifically the bilateral alternation of the same muscles (IP L vs. IP R, TA L vs. TA R and GM L vs. GM R). The significant values of the ground reaction forces also indicate the stable reciprocal activity of the extensor muscles (Fig. 2). The temporal patterns of muscle activation based on the mean EMG data are shown in Fig. 3. The ankle and hip flexors (TA and IP, respectively) were active during the swing and the ankle extensors (GM) were active during the stance for each locomotor mode. Again, the pattern of muscle activation during FW_BIDI hindlimb stepping was more like FW, and the pattern of muscle activation during BW_BIDI hindlimb stepping was more like BW. The activity of the abovementioned flexors relative to FW was significantly lower during BW and BW_BIDI hindlimb locomotion [W(14) = 103, *p* = 0.0002 and W(7) = 28, *p* = 0.0156 respectively] (Fig. 4b,c). The activity of extensors relative to FW was significantly lower during FW_BIDI hindlimb locomotion [W(7) = 28, *p* = 0.0156] (Fig. 4a). Thus, the well-coordinated locomotor movements were generated by each limb during BIDI stepping with bilateral alternation and specific reciprocal modulation between antagonist muscles despite walking in different directions.

**Figure 5:**
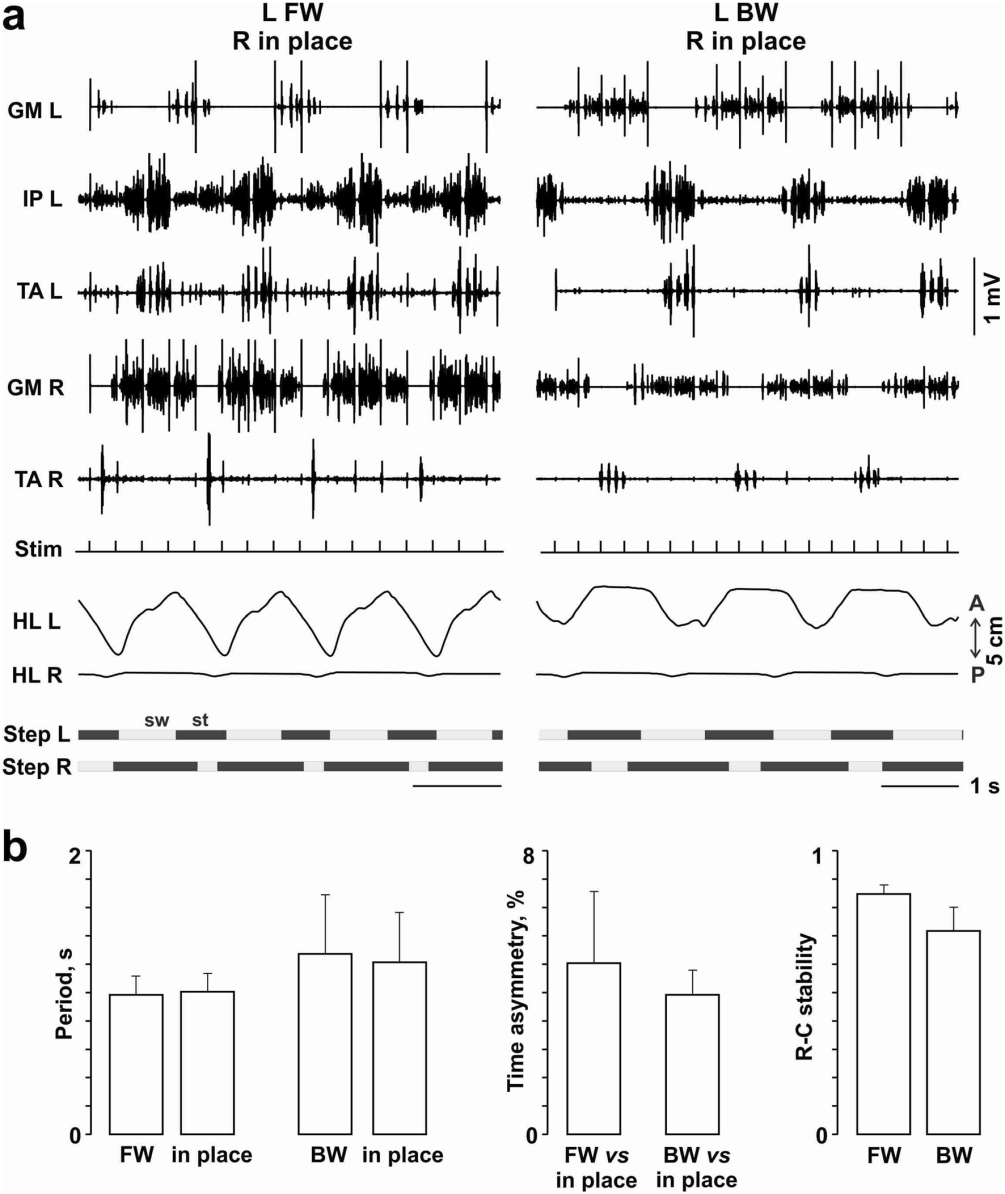
FW or BW of one leg while the other leg is stepping in place. (**a**) EMG pattern of left and right mm. iliopsoas (IP), tibialis anterior (TA) and gastrocnemius medialis (GM) during subsequent stepping of left and right hindlimbs (HL L, HL R). (**b**) The step cycle period and its asymmetry and the rostrocaudal stability.

To investigate *the strength of commissural connectivity* we performed supplementary experiments that included stopping one of two belts while the speed of the other belt remained constant (Fig. 5). Only 2 of 4 animals tested were able to perform coordinated FW or BW of one leg while the other leg tapped the stopped belt in place. The EMG responses of hindlimb muscles stepping in place depended on the other leg’s walking direction (Fig. 5a). The locomotor characteristics were qualitatively similar to those received for normal FW and BW. The step cycle of both hindlimbs during one leg FW was shorter than the step cycle of both hindlimbs during one leg BW, and the FW rostrocaudal stability was higher than that of BW (Fig. 5b). The step cycle asymmetry was relatively low in both conditions.

**Figure 6:**
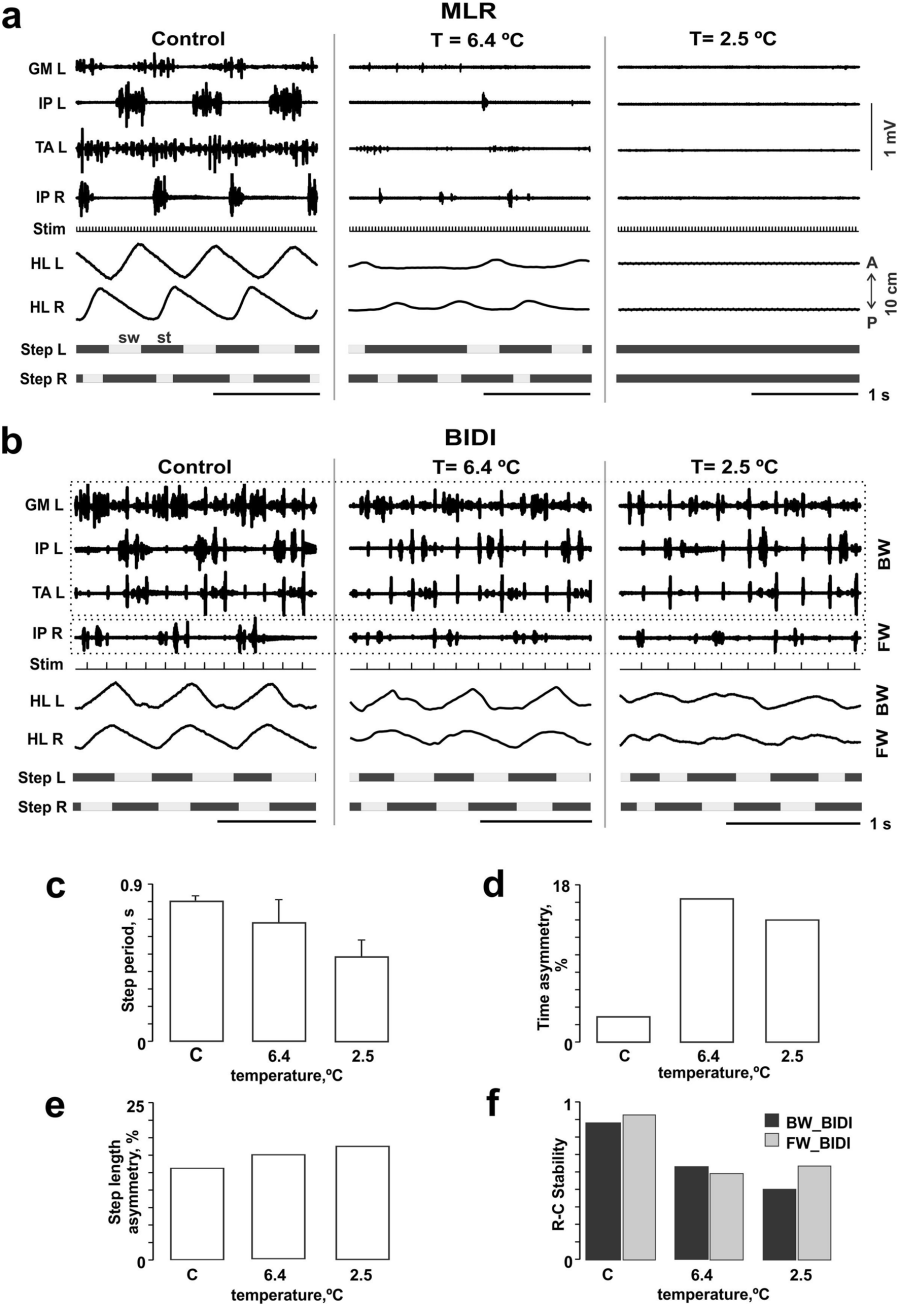
Temporary cooling of the spinal cord dorsal surface as a simulation of spinalization. (**a**,**b**) EMG patterns of left and right mm. iliopsoas (IP), tibialis anterior (TA) and gastrocnemius medialis (GM) during subsequent stepping of left and right hindlimbs (HL L, HL R) at mesencephalic locomotor region (MLR) evoked FW versus BIDI locomotion at different temperatures. (**c**–**f**) The step cycle period and its asymmetry, step length asymmetry and the rostrocaudal stability at different temperatures (BW_BIDI means hindlimb moving backward, FW_BIDI means hindlimb moving forward during BIDI stepping).

To check *the supraspinal influence on the BIDI locomotion*, we simulated the spinalization by temporary cooling of the dorsal surface of the T13 segment in one animal (Figs. 1a and 6). Before cooling (control), the animal was capable of FW with MLR stimulation and of BIDI locomotion with ES. It is worth noting that we failed to induce BW with MLR stimulation, as was also seen in a previous study^27^; this locomotor mode may be invoked only with ES of the L5-L7 trigger zone^21^. BIDI locomotion was also absent with MLR stimulation. At cooling down to 6.4 °C, the quality of MLR-evoked locomotion significantly decreased (Fig. 6a). At further cooling down to 2.5 °C, MLR stimulation could not elicit locomotion due to blockage of the descending spinal tracts in the ventral spinal cord^26,28^. BIDI locomotion was still possible, though its quality worsened at the same temperature (Fig. 6b). In general, the asymmetry of the step length and period (Fig. 6c–e) increased with a decrease in temperature, while the step period and the rostrocaudal stability of both hindlimbs decreased (Fig. 6f).

## Discussion

### Bidirectional stepping phenomenon

The main finding of the present study was that ES evoked bidirectional stepping in decerebrated animals with no forebrain control upon the spinal locomotor networks. We analysed basic features of BIDI locomotion: EMG activation pattern in flexor and extensor muscles, as well as main kinematic characteristics. The data point to BIDI stepping as a stable and well-coordinated mode comparable to unidirectional stepping. Simultaneous bidirectional locomotion in decerebrated animals confirms that the neuronal circuitry of the spinal cord, brainstem, and cerebellum is enough to control such highly coordinated and extraordinary locomotor tasks.

Activity of the spinal locomotor networks is dependent upon supraspinal input. At first, supraspinal input regulates a general level of the spinal excitability^29^. Second, Mitchell et al.^30^ had shown strong reticulospinal connections with the commissural interneurons in the rat that suggests the supraspinal control of the left–right coordination. In any case, cooling disrupts the supraspinal influence on the spinal networks and can be a reason for the deterioration of the BIDI stepping. However, the preliminary data show that BIDI locomotion is possible during temporary cooling, although its quality significantly decreases (Fig. 6). This presents evidence for the great degree of automatism involved in BIDI stepping defined by the spinal neuronal networks.

Yang et al. found that adult humans rarely use BIDI locomotion and concluded that the mechanisms of BIDI locomotion in humans are in some sense rudimental^17^. This may be the reason that they succeeded in inducing BIDI locomotion in only 6 out of 10 infants, compared to our ability to induce BIDI locomotion in all 7 of our decerebrate animals. Thus, the decerebrate cat is a good model for studying the different locomotor modes.

### Commissural interconnections

It is well known that all events of the hindlimb step during typical diagonal walking are shifted approximately half a cycle up to the corresponding events on the contralateral side^31^. The present work shows that this pattern is preserved when hindlimbs are forced to move in opposite directions. This supports the hypothesis of the multilevel CPG^5^, including levels of rhythm generation and pattern formation. The presence of the former follows from a stable rhythmic walking. The presence of the latter follows from the correct flexor and extensor muscle activity of both hindlimbs despite the different joint angles due to different belt directions (Fig. 1c).

During ES the specific somatosensory input can change the direction mode of CPGs independently for the left and right sides. On the contrary, both BW and BIDI locomotion are absent during MLR stimulation, although its stimulation evokes FW. This confirms that the reciprocal muscle activity during locomotion generated by CPG depends on the supraspinal drive as well as on the somatosensory input. Presumably, the regulation of the duration of stance and swing phases is performed independently by FW_BIDI and BW_BIDI hindlimbs (Fig. 1m). At the same time, the commissural interconnections regulate the temporal shift between the cycles of the left and right CPGs. Due to such interconnections, the step periods for both hindlimbs are approximately equal in BIDI locomotion (Fig. 1l) which gives evidence of strong commissural communication in both unidirectional and bidirectional locomotion. A similar finding was obtained for the split-belt paradigm for FW; for some intervals of split-belt speeds the speeds of both legs of infants^13^ or both hindlimbs of spinal cats^12^ moving on fast and slow belts were equal and intermediate between the belt speeds.

We additionally assessed the inter-limb coupling by distortion of the sensory input to the one hindlimb when the treadmill belt was stopped on the same side. Even in this case, 2 out of 4 cats were able to continue the bilateral rhythmical reciprocal locomotor activity despite one limb performing a FW or BW stepping action and the other limb stepping in place. The background excitability of the brainstem and the spinal cord varies between cats^32^ and thereby can be a reason for the successful or unsuccessful locomotion. Possibly, this was the same reason for the different ability of the animals studied to continue locomotion after one treadmill belt stopped. The step period of both hindlimbs was equal, proving that the neuronal mechanisms of bilateral coordination were functional (Fig. 5).

### Comparison of unidirectional and bidirectional stepping

We have shown here that during BIDI locomotion, each hindlimb follows the ‘programme’ of the corresponding unidirectional locomotion. It was illustrated by significant differences between two hindlimbs during BIDI mode, comparable to the differences between unidirectional FW and BW. The data obtained are in agreement with previous studies^21,24^. Both for unidirectional and bidirectional mode, the locomotor cycle of the limb stepping BW was significantly larger than that of FW due to the increase in the swing phase. Presumably, this difference originated from the level of the flexors and extensors activity. Since Sherrington’s study^33^ it has been well known that flexor activity is greatly facilitated by the hip extension. Conversely, it may be inhibited by the hip flexion. The degree of hip flexion may be the reason for the revealed differences between the FW and BW modes. In the beginning of the swing phase, during BW, the hip is flexed to a greater degree than during FW. Thus, we suggest that the duration of the swing phase as well as the step length depends not only on the treadmill speed^31^ but also on the position of the hip in the beginning of the swing phase. Hence, the decreased activity of the flexors (TA, IP) during BW and BW_BIDI compared to FW and FW_BIDI stepping obtained here may be explained by the more flexed position of the hip at the beginning of the swing phase; the major flexion was performed in the knee joint. The activity of the ipsilateral flexors and contralateral extensors was positively correlated due to their mutual excitatory interconnections^34^. That could be one of the mechanisms underlying reduction activity of the GM in the FW_BIDI hindlimb led by the decreased activity of the TA and IP in the BW_BIDI hindlimb (Fig. 4).

To conclude, we have shown that the decerebrate animals were capable of BIDI locomotion on a split-belt treadmill with equal periods of stepping for both hindlimbs. The kinematic characteristics of the FW_BIDI hindlimb and BW_BIDI hindlimb were similar to the features of the corresponding unidirectional FW and unidirectional BW stepping. The interlimb coordination during FW and BW was preserved in 2 out of 4 animals at ‘extreme’ conditions when one of the treadmill belts was stopped. This means that the neuronal circuitries of the spinal cord, brainstem, and cerebellum are enough to control such locomotor tasks. The temporary spinalization degrades the quality of BIDI locomotion; however, the possibility of BIDI locomotion is saved. This presents evidence for the great degree of automatism for this stepping mode defined by the spinal neuronal networks.

## Supplementary Information

Supplementary Information 1.

Supplementary Video 1.

## Data Availability

The raw and analysed datasets are available for research purposes from the corresponding author on reasonable request.
