# Supplementary material for: Simultaneous bidirectional hindlimb locomotion in decerebrate cats
Source: Sci Rep. 2021 Feb 5;11:3252. doi: 10.1038/s41598-021-82722-2 (PMC7865075; doi:10.1038/s41598-021-82722-2)
Supplement: Supplementary file 1 — Supplementary Information 1. [file 41598_2021_82722_MOESM1_ESM.docx]

Supplementary materials for:

**Simultaneous bidirectional hindlimb locomotion in decerebrate cats**

Lyakhovetskii V.^1,2^, Merkulyeva N.^1,2,3^, Gorskii O.^1,2,3^, *Musienko P ^2,3,4^

*^1^Russian Research Center of Radiology and Surgical Technologies, Ministry of Healthcare of the RF, poselok Pesochnyy, Leningradskaya st., 70, Saint-Petersburg, 197758, Russia*

*^2^Pavlov Institute of Physiology, Russian Academy of Sciences, emb. Makarova 6, Saint-Petersburg, 199034, Russia*

*^3^Institute of Translational Biomedicine, Saint-Petersburg State University, Saint-Petersburg, Universitetskaya emb. 7/9, 199034, Russia*

*^4^Children’s Surgery and Orthopedic Clinic, Saint-Petersburg State Research Institute of Phthisiopulmonology, Ministry of Healthcare of the RF, Saint-Petersburg, 191036, Russia*

***Correspondence**: Pavel Musienko: e-mail: [pol-spb@mail.ru](mailto:pol-spb@mail.ru)

**Multimedia**

***Supplement Video.*** *Decerebrate cat stepping forward, backward and bidirectional during epidural stimulation.*
